# Serum Glutathione and Malondialdehyde Levels as Predictors of Early Neurological Deficits and Short-Term Outcomes in Acute Cerebral Infarction

**DOI:** 10.33549/physiolres.935544

**Published:** 2025-04-01

**Authors:** Yu-Xi WANG, Jing-Ying WANG, Hui YANG, Rui ZHANG, Rong CAO, Wei HONG, Su JIANG

**Affiliations:** 1 Department of Neurology, The Affiliated Taizhou People’s Hospital of Nanjing Medical University, Taizhou School of Clinical Medicine, Nanjing Medical, Taizhou, China; 2 School of Nursing, Nanjing University of Chinese Medicine, Nanjng, China; 3 Department of Respiratory, The Affiliated Taizhou People’s Hospital of Nanjing Medical University, Taizhou School of Clinical Medicine, Nanjing Medical, Taizhou, China; 4 Department of Rehabilitation, The Affiliated Taizhou People’s Hospital of Nanjing Medical University, Taizhou School of Clinical Medicine, Nanjing Medical, Taizhou, China

**Keywords:** Acute cerebral infarction, Malondialdehyde, Neurological deficits, Serum glutathione, Short-term prognosis

## Abstract

This study investigates the association between serum glutathione (GSH) and malondialdehyde (MDA) levels and early neurological deficits and short-term outcomes in individuals with acute cerebral infarction (ACI). The study included 114 patients with ACI within 48 hours of symptom onset, between January and August 2023, alongside 96 healthy individuals as a control group. Neurological deficits were assessed using the National Institute of Health Stroke Scale (NIHSS), classifying deficits as mild (<5) or moderate to severe (≥5). Associations between GSH and MDA levels with early neurological deficits were analyzed. Short-term prognosis, assessed three months post-discharge using the Modified Rankin Scale (mRS), was examined in relation to GSH and MDA levels in patients with ACI. Independent predictors of neurological deficits and short-term outcomes were identified through binary logistic regression analysis. Compared to the control group, patients with ACI had higher rates of hypertension, diabetes, smoking, and alcohol consumption. Additionally, elevated levels of MDA, glycated hemoglobin, triglycerides, C-reactive protein (CRP), and D-dimer levels were observed, whereas GSH and high-density lipoprotein (HDL) levels were lower. Among those with moderate to severe ACI, levels of CRP, MDA, triglycerides, low-density lipoprotein (LDL), uric acid, and D-dimer levels were higher compared to mild ACI, while HDL and GSH levels were significantly lower. Low serum GSH levels and elevated MDA levels are associated with early neurological deficits and short-term prognosis in ACI, serving as independent risk factors for adverse prognosis. The combined assessment of MDA, infarct volume, and LDL provides enhanced predictive value for adverse prognosis in patients with ACI.

## Introduction

Stroke remains the leading cause of mortality in China, with cerebral infarction accounting for approximately 69.6 % to 70.8 % of all stroke cases. This prevalence poses a serious threat to patient health and imposes a significant burden on society [1]. Ferroptosis, a recently identified form of cell death has been shown to play a crucial role in neurological diseases and ischemia-reperfusion injury [2,3]. During ischemic events, impaired cerebral blood flow increases transferrin and transferrin receptor expression, resulting in elevated iron levels in brain tissue [4]. Excessive iron accumulation in brain tissue generates reactive oxygen species (ROS) through the Fenton reaction, leading to lipid peroxidation and the production of byproducts such as malondialdehyde (MDA) [5]. This oxidative process promotes the reaction of cellular and tissue proteins or DNA to form adducts, resulting in biomolecular damage. Serum glutathione (GSH) is a key antioxidant that plays a protective role by mitigating oxidative stress and reducing cellular damage from ROS attacks. According to research, patients with a history of cerebral infarction tend to have lower GSH levels [6].

This study aims to examine the differences in serum GSH and MDA levels among individuals with acute cerebral infarction (ACI) in relation to early neurological deficits and short-term prognosis. It further seeks to evaluate the potential of these biomarkers as reliable indicators for assessing disease severity and predicting short-term outcomes in ACI.

## Methods

### Participants and methods

#### Study participants

This retrospective analysis included 114 patients with ACI admitted to the Neurology Department of the Affiliated Taizhou People’s Hospital of Nanjing Medical University between January and August 2023, all presenting within 48 hours of symptom onset. Additionally, the control group consisted of 96 healthy individuals who underwent routine health examinations at the hospital’s health check-up center during the same period. Informed consent was obtained from all participants or their legal guardians. The study was approved by the Ethics Committee of the Affiliated Taizhou People’s Hospital of Nanjing Medical University (Approval No. KY 2023-111-01).

#### Inclusion and exclusion criteria

Inclusion criteria for ACI group:

- Diagnosis consistent with the “acute ischemic stroke” criteria is outlined in the *Guidelines for the Diagnosis and Treatment of Acute Ischemic Stroke in China 2018* with clear infarct lesions visible on computed tomography (CT) or magnetic resonance imaging (MRI) and no evidence of hemorrhage [7].
- No vascular recanalization treatments, such as thrombolysis or thrombectomy, performed.
- Age between 18 and 80 years.
- Signed informed consent was provided.

Exclusion criteria for ACI group:

- Patients who have undergone vascular recanalization treatments, such as intravenous thrombolysis or thrombectomy.
- Presence of liver or kidney dysfunction, tumors, heart failure, or clear infection upon admission.

Control group inclusion criteria:

- Absence of clinical symptoms with ischemic stroke, with no visible lesions observed on cranial MRI.

Control group exclusion criteria:

- Presence of liver or kidney dysfunction, tumors, heart failure, or a clear infection indicated by laboratory tests.

#### Severity grouping

Severity classification for ACI was based on the National Institute of Health Stroke Scale (NIHSS). Patients with an NIHSS score below 5 are classified as having mild ACI, while those with a score of 5 or higher are categorized in the moderate-to-severe group.

#### Prognosis grouping

Prognostic classification at 90 days post-discharge was determined using the Modified Rankin Scale (mRS). Patients with an mRS score of 2 or below are assigned to the good prognosis group, whereas those with an mRS score above 2 are assigned to the poor prognosis group.

#### Observation indicators

Baseline data for all study participants, including demographic information (gender, age) and clinical history (hypertension, diabetes, coronary heart disease, smoking, and alcohol use), along with NIHSS score at admission, were obtained from the Hospital Information System at the Affiliated Taizhou People’s Hospital of Nanjing Medical University. Laboratory indicators such as glycosylated hemoglobin, triglycerides, total cholesterol, high-density lipoprotein, low-density lipoprotein, uric acid, C-reactive protein, and D-dimer were collected using the Laboratory Information Management System. Cranial MRI data for all patients in the ACI group were sourced from the Picture Archiving and Communication System. The lesion volume was calculated using the simplified Pullicino formula: (ACI lesion longest diameter × widest diameter × (slice thickness + slice spacing) × number of positive slices) ÷ 2. Follow-up assessments were conducted via phone to determine the mRS score three months post-discharge.

### Experimental methods

#### Sample collection and processing

Fasting blood samples were collected from patients with ACI within 24 hours of admission and again on the morning of the seventh day post-onset. Fasting blood samples for the control group were also collected within 24 hours. The samples were centrifuged at 3,000 rpm for 10 minutes, and the supernatant was preserved at −80 °C.

#### Serum sample testing

**Fig. 1:**
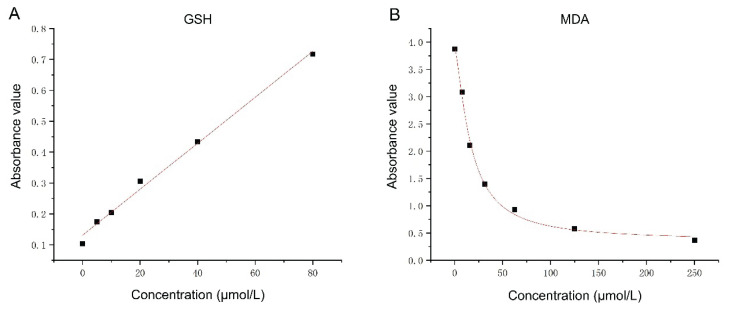
**A:** Standard curve for glutathione (GSH) levels; **B:** Standard curve for malondialdehyde (MDA) levels. x axis: Concentration (μmol/L); y axis: Absorbance value.

Serum concentrations were determined using enzyme-linked immunosorbent assay (ELISA) kits. Standard curves and equations (Fig. 1) were generated to calculate serum concentrations. The ELISA kit for reduced GSH was purchased from Wuhan Yipu Biotechnology Co., Ltd., and the kit for MDA was purchased from Nanjing Juxionghua Biotechnology Co., Ltd.

#### Statistical methods

All statistical analyses were conducted using SPSS software (version 26) and R software (version 4.3.1). Sample size calculations for the predictive model were conducted using the pmsampsize package in R (version 4.3.1) [8]. Based on similar research by Heo *et al*. the C statistic is set at 0.849 [9]. According to current global epidemiological studies, the incidence of stroke ranges from approximately 24.9 to 39.3 % [10]. This study considered 15 candidate predictive variables. The syntax used for calculation is: pmsampsize (type = “b”, cstatistic = 0.849, parameters = 15, prevalence = 0.393), yielding a required sample size of approximately 200 to 367 individuals.

For measurement data that follow a normal distribution, results were presented as (x ± s); for data that do not follow a normal distribution, results were presented as the median and interquartile range [M (P25, P75)]. Enumeration data were expressed as percentages (n, %). Comparisons between two groups used a two-sample *t*-test for normally distributed data and the Mann-Whitney U test for data that are not normally distributed. Enumeration data was represented as [n (%)]. and analyzed using the chi-squared test or Fisher’s exact probability test.

A binary logistic regression model was constructed, with variables showing variance inflation factor values exceeding 10 excluded from the model. Model validation and construction followed this selection process.

## Results

### Comparison of baseline data and clinical characteristics

Table 1 presents the baseline data for 114 cases in the ACI group and 96 in the control group. No statistically significant differences were observed between the groups in terms of age and gender (*P*>0.05). However, compared to the control group, the ACI group had a significantly higher proportion of individuals with a history of hypertension, diabetes, smoking, and alcohol use with statistically significant differences (*P*<0.05). Additionally, GSH levels were significantly lower in the ACI group (*P*<0.05), while MDA levels were significantly higher (*P*<0.05). Laboratory indicators such as glycosylated hemoglobin, triglycerides, high-density lipoprotein, uric acid, C-reactive protein, and D-dimer were also elevated in the ACI group (P<0.05).

### Analysis of serum GSH and MDA levels and ACI severity

As shown in Table 2, the ACI group of 114 patients was divided into a mild group (65 cases) and a moderate-to-severe group (48 cases) based on NIHSS scores at admission. No statistically significant differences were observed in the baseline data such as age, gender, smoking, alcohol use, histories of hypertension and diabetes across the severity groups (*P*>0.05). However, infarct volume, triglycerides, high-density lipoprotein, low-density lipoprotein, uric acid, C-reactive protein, and D-dimer exhibited significant differences (*P*<0.05). GSH levels were significantly higher in the mild group, compared to the moderate-to-severe group (*P*<0.05), whereas serum MDA level concentrations were significantly lower in the mild group compared to the moderate-to-severe group at admission (*P*<0.05).

### Analysis of serum GSH and MDA levels and ACI prognosis

Table 3 presents the data based on the mRS scores at three months post-ACI, dividing patients into a good prognosis group (71 cases) and a poor prognosis group (42 cases). No statistically significant differences in the baseline data were observed between the prognosis groups (*P*>0.05). However, infarct volume, low-density lipoprotein, C-reactive protein, and D-dimer levels were significantly lower in the good prognosis group (*P*<0.05). Serum GSH levels were significantly higher in the good prognosis group compared to the poor prognosis group (*P*<0.05), while serum MDA levels were significantly lower in the good prognosis group (*P*<0.05).

### Factors influencing poor prognosis in patients with ACI: binary logistic regression analysis

Initial concentrations of serum GSH and MDA, infarct volume, low-density lipoprotein, C-reactive protein, and D-dimer were identified as significant factors influencing prognosis in patients with ACI (Table 4). Binary logistic regression analysis including these risk factors revealed that serum MDA concentration, infarct volume, and low-density lipoprotein were independent risk factors for poor prognosis in patients with ACI (*P*<0.05).

### ROC curve analysis

**Fig. 2:**
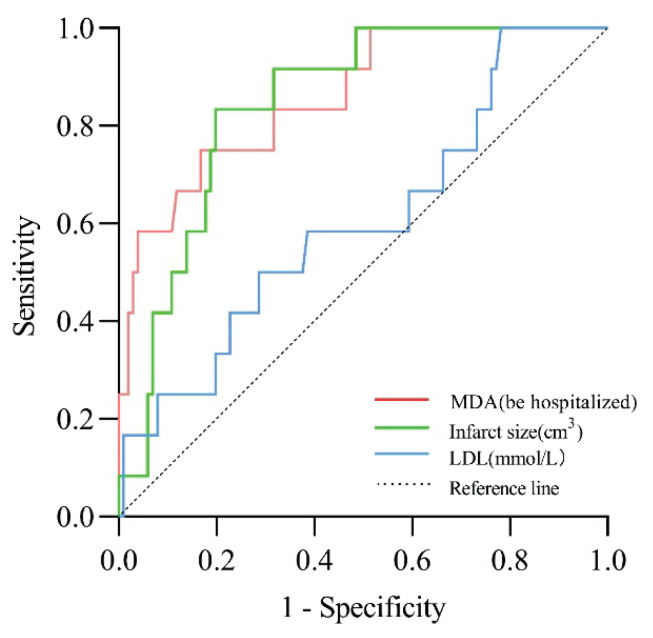
Predictive value analysis of serum MDA, infarct volume, low-density lipoprotein (LDL), and combined biomarkers for prognostic assessment in patients with acute cerebral infarction (ACI).

The ROC curve analysis indicated that the combined indicators of serum MDA, infarct volume, and low-density lipoprotein achieved an area under the curve (AUC) of 0.784 in predicting poor prognosis in patients with ACI (*P*<0.05, 95 % CI: 0.692–0.876) (Fig. 2, Table 5).

## Discussion

ACI is a major contributor to global mortality and disability, creating significant psychological and economic burdens on both society and affected families. Advances in technology have established endovascular recanalization treatments such as intravenous thrombolysis and endovascular thrombectomy as essential approaches for ACI management. However, a significant number of patients remain ineligible for timely recanalization therapy due to either presentation outside the therapeutic window or contraindications to treatment [11]. Thus, early prediction of ACI severity and prognosis is crucial for timely assessment and treatment adjustments.

Recent research has highlighted the role of ferroptosis in various pathological conditions, including oncological, neurological, renal, and ischemia-reperfusion injuries. Ferroptosis is primarily regulated through mechanisms involving iron overload, lipid peroxidation, and an imbalance in antioxidant systems like the glutathione/glutathione peroxidase 4 (GSH/ GPX4) axis [12–15]. MDA is a significant degradation product of lipid peroxidation, which increases membrane permeability and induces oxidative damage [16]. The accumulation of degradation products is a defining feature of ferroptosis. GSH, a vital antioxidant in human cells, helps minimize the accumulation of lipid peroxides and ROS, thereby inhibiting ferroptosis [17].

Following cerebral infarction, disruption of the blood-brain barrier increases permeability, permitting iron to infiltrate brain parenchyma and resulting in iron overload. GSH can bind to excess iron in the brain, thereby reducing iron levels and mitigating oxidative damage from lipid peroxides and ROS [18]. Furthermore, GSH scavenges ROS generated during ischemia-reperfusion injury, thereby reducing excessive oxidative stress and subsequent cellular damage. Lipid peroxidation reaction continuously produces lipid hydroperoxides (LOOH), which, in the presence of ferrous ions, initiate lipid peroxidation, a key step in ferroptosis. GPX4, in the presence of GSH, decomposes LOOH into non-toxic byproducts, thereby inhibiting the initiation of lipid peroxidation and reducing cellular death.

Research indicates that iron deposition in brain tissue among patients with cerebral infarction may exacerbate ferroptosis in ischemic neurons, leading to GSH depletion and worsening clinical symptoms [19,20]. After GSH is depleted, ROS and LOOH cannot be effectively neutralized, resulting in intensified lipid peroxidation reactions. The accumulating lipid peroxides damage cellular proteins and DNA, ultimately leading to cell death, which exacerbates brain tissue damage and increases infarct volume [21]. This negatively impacts the severity of the condition and worsens the prognosis for patients with ACI.

The current study corroborated this perspective, with a comparative analysis revealing serum GSH levels of 223.18 ng/L in healthy controls, contrasted with levels of 58.39 ng/L in patients with ACI within 48 hours post-onset. These levels gradually recovered to 122.89 ng/L after the acute phase. This fluctuation may be related to the significant generation of strong oxidants, such as ferrous ions and ROS, following the ischemia-reperfusion injury in cerebral infarction, which results in considerable GSH depletion. As the acute phase subsides and lipid peroxidation reactions diminish, GSH levels recover gradually, reducing the accumulation of lipid peroxides and lowering the risk of symptom progression, thereby facilitating recovery. Subsequent serum tests conducted seven days post-onset indicated that GSH levels in patients with poor prognosis remained significantly lower than those in patients in the good prognosis group, while the MDA levels were significantly higher. These findings suggest that elevated ischemia-reperfusion injury and diminished oxidative stress defenses following ACI may heighten the risk of poor prognosis in patients.

Research has suggested that low GSH levels may induce ferroptosis in neurons, thereby increasing the risk of severe neurological deficits [22]. A study by Wang *et al*. indicated that oral administration of exogenous GSH exerts therapeutic effects, either directly or indirectly, on mice with ACI. Additional studies on post-stroke reperfusion mechanisms revealed that exogenous GSH administration increased dopamine levels, providing therapeutic benefits for ischemic stroke [23]. Similarly, findings by Song *et al*. demonstrated that treatment with exogenous GSH significantly reduced the infarct volume in ACI mice [24].

The results of the current study also presented a correlation between the degree of neurological deficits in patients with ACI and GSH levels. Data indicated significantly lower serum GSH levels in patients with moderate-to-severe ACI compared to those with mild presentations, while serum MDA levels were significantly higher in the moderate-to-severe group. This pattern likely reflects excessive depletion of GSH, leading to unregulated oxidative stress responses. The continuous accumulation of lipid peroxides and ROS may lead to extensive brain tissue damage, increased infarct volume, and exacerbated neurological deficits in affected individuals.

This study identified serum MDA levels, infarct volume, and low-density lipoprotein (LDL) as independent risk factors for poor prognosis in patients with ACI through binary logistic regression analysis. The ROC curve analysis indicated that the AUC values for MDA, infarct volume, and LDL at the early stage of onset were 0.768, 0.759, and 0.614, respectively. Notably, an initial MDA concentration of ≥ 22.25 ng/ml was associated with a markedly higher risk of poor prognosis among patients with ACI. The combined assessment of MDA, infarct volume, and LDL enhances the predictive accuracy for poor prognosis in ACI, offering valuable prognostic insight. Our findings demonstrated that combining MDA with infarct volume and LDL levels provides enhanced predictive value (AUC 0.784), supporting a multi-parameter assessment strategy, and providing a more balanced perspective on the utility of these biomarkers in ACI assessment while acknowledging their limitations. These biomarkers should be interpreted as complementary tools alongside established clinical and imaging parameters, rather than standalone diagnostic indicators. This was well demonstrated by the improved predictive value (AUC 0.784) when combining MDA with other clinical parameters. We had also included a more critical analysis of the oxidative stress pathway in ACI, acknowledging that these markers reflect part of a complex pathophysiological cascade rather than serving as specific diagnostic indicators.

The sample size of 114 ACI patients may limit the generalizability of our conclusions. While the results align with the existing literature, we believe they contribute meaningful validation data in an Asian population and provide important insights into the combined utility of these biomarkers with conventional clinical parameters. To address this limitation, we have conducted post-hoc power analyses and included a detailed discussion of potential sampling bias. Most importantly, despite the inherent variability in biomarker levels, the observed correlations remained statistically significant after adjusting for potential confounders.

## Conclusion

In conclusion, GSH and MDA are supporting a multi-parameter assessment strategy, and providing a more balanced perspective on the utility of these biomarkers in ACI assessment. They should be interpreted as complementary tools alongside established clinical and imaging parameters, rather than standalone diagnostic indicators. These markers reflect part of a complex pathophysiological cascade rather than serving as specific diagnostic indicators. However, this study has certain limitations. As a single-center study with a limited sample size, there could be constraints in the statistical power and potential bias in the findings. Blood samples were collected only at onset and on the seventh day post-infarction, providing limited data points. Future researches should include dynamic monitoring of serum biomarkers over extended periods, ranging from 1 to 3 months or more, to further elucidate their correlation with rehabilitation outcomes. Additionally, our findings serve as a foundation for larger, multi-center studies to further validate these associations.

## Figures and Tables

**Table 1:** Comparison of baseline clinical characteristics between individuals with ACI and the healthy control group

	Cerebral Infarction Group (n = 114)	Healthy Control Group (n = 96)	*P* value
***Baseline Characteristics***			

*Age (years)*	68.03±10.67	62.02±8.41	0.104
*Male participants, n (%)*	71 (62.8)	48 (51.1)	0.150
*Hypertension, n (%)*	87 (77)	43 (45.7)	0.000
*Diabetes, n (%)*	51 (45.1)	20 (21.3)	0.000
*Smoking History, n (%)*	35 (31)	12 (12.8)	0.002
*Drinking History, n (%)*	30 (26.5)	12 (12.8)	0.014
*Glycated Hemoglobin (mmol/L)*	6.40 (5.80, 7.57)	6.01 (5.60, 6.40)	0.003
*Triglycerides (mmol/L)*	1.39 (0.91, 1.80)	1.19 (0.79, 1.12)	0.031
*Total Cholesterol (mmol/L)*	4.44±1.03	1.30±0.68	0.067
*High-Density Lipoprotein (mmol/L)*	1.10 (0.93, 1.23)	1.20 (0.98, 1.41)	0.004
*Low-Density Lipoprotein (mmol/L)*	1.09±0.27	2.94±0.91	0.146
*C-Reactive Protein (mg/L)*	1.89 (0.75, 5.33)	0.77 (0.55, 0.85)	0.000
*D-Dimer (μg/L)*	0.73 (0.56, 1.71)	0.59 (0.44, 0.85)	0.003
***Observational Indicators***			

*Glutathione (ng/L)*	58.39 (22.44, 97.81)	223.18 (172.94, 277.29)	0.000
*Malondialdehyde (ng/ml)*	16.72 (11.26, 22.56)	1.44 (1.31, 1.54)	0.000

**Table 2:** Comparative analysis of serum GSH and MDA levels in patients with ACI according to disease severity

	Mild (n = 65)	Moderate to Severe (n = 48)	*P* value
*Age (years)*	69.00±10.81	70.00±10.36	0.327
*Male participants, n (%)*	43 (66.2)	26 (54.2)	0.198
*Hypertension, n (%)*	48 (73.8)	39 (81.3)	0.357
*Diabetes, n (%)*	30 (46.2)	21 (43.8)	0.327
*Smoking History, n (%)*	18 (27.7)	17 (35.4)	0.382
*Drinking History, n (%)*	14 (21.5)	16 (33.3)	0.162
*Glycated Hemoglobin (mmol/L)*	6.40 (5.75, 6.85)	6.15 (5.83, 8.39)	0.905
*Triglycerides (mmol/L)*	1.20 (0.88, 1.59)	1.55 (1.09, 2.08)	0.013
*Total Cholesterol (mmol/L)*	4.30±0.98	4.56±1.11	0.195
*High-Density Lipoprotein (mmol/L)*	1.14 (1.10, 1.29)	1.02 (0.90, 1.19)	0.032
*Low-Density Lipoprotein (mmol/L)*	2.68±0.70	2.85±0.04	0.039
*Uric Acid (μmol/L)*	341.00 (298.00, 416.00)	383.00 (319.25, 457.00)	0.011
*C-Reactive Protein (mg/L)*	1.46 (0.63, 3.04)	3.76 (1.65, 9.61)	<0.001
*D-Dimer (μg/L)*	0.65 (0.54, 1.17)	1.09 (0.66, 3.01)	<0.001
*Glutathione (upon admission) (ng/L)*	68.46 (44.65, 109.96)	36.64 (15.03, 70.14)	0.006
*Glutathione (Day 7) (ng/L)*	126.80 (71.81, 165.23)	86.44 (46.34, 129.62)	<0.001
*Malondialdehyde (upon admission) (ng/ml)*	13.06 (9.05, 18.93)	21.32 (15.35, 35.27)	<0.001
*Malondialdehyde (Day 7) (ng/ml)*	7.19 (4.41, 12.24)	15.01 (9.66, 29.73)	<0.001
*Infarct Volume (cm* *^3^* *)*	1.09 (0.43, 3.26)	11.24 (2.94, 27.41)	<0.001

**Table 3:** Comparative analysis of serum GSH and MDA levels in patients with ACI based on prognosis

	Good Prognosis Group (n = 71)	Poor Prognosis Group (n = 42)	*P* value
*Age (years)*	70.00±10.43	70.00±10.88	0.250
*Hypertension, n (%)*	52 (73.2)	35 (83.3)	0.220
*Diabetes, n (%)*	30 (42.3)	21 (50.0)	0.426
*Smoking History, n (%)*	22 (31.0)	13 (31.0)	0.997
*Drinking History, n (%)*	19 (26.8)	11 (26.2)	0.947
*Glycated Hemoglobin (mmol/L)*	6.30 (5.70, 7.10)	6.40 (5.90, 8.86)	0.353
*Triglycerides (mmol/L)*	1.36 (0.87, 1.74)	1.43 (1.12, 1.90)	0.089
*Total Cholesterol (mmol/L)*	4.30±0.95	4.71±1.16	0.140
*High-Density Lipoprotein (mmol/L)*	1.12 (0.94, 1.28)	1.06 (0.91, 1.19)	0.199
*Low-Density Lipoprotein (mmol/L)*	2.66±0.66	2.93±0.70	0.044
*Uric Acid (μmol/L)*	351.00 (293.00, 244.00)	376.50 (323.75, 443.50)	0.079
*C-Reactive Protein (mg/L)*	1.52 (0.65, 3.51)	3.76 (1.56, 9.39)	<0.001
*D-Dimer (μg/L)*	0.71 (0.54, 1.42)	0.99 (0.66, 1.89)	0.028
*Glutathione (upon admission) (ng/L)*	62.72 (35.54, 104.75)	41.92 (14.29, 73.17)	0.004
*Glutathione (Day 7) (ng/L)*	122.89 (71.80, 165.23)	83.92 (46.13, 130.95)	0.006
*Malondialdehyde (upon admission) (ng/ml)*	13.49 (9.33, 18.92)	22.53 (15.24, 36.52)	<0.001
*Malondialdehyde (Day 7) (ng/ml)*	7.66 (4.89, 13.04)	16.01 (9.04, 29.83)	<0.001
*Infarct Volume (cm* *^3^* *)*	1.27 (0.48, 5.66)	8.34 (2.19, 30.66)	<0.001

**Table 4:** Binary logistic regression analysis of factors affecting prognosis in individuals with cerebral infarction

Variable	OR (95 % CI)	P value
*Glutathione (upon admission) (ng/L)*	0.991 (0.981, 1.001)	0.073
*Malondialdehyde (upon admission) (ng/ml)*	1.036 (1.004, 1.069)	0.026
*Infarct Volume (cm* *^3^* *)*	1.032 (1.002, 1.064)	0.037
*Low-Density Lipoprotein (mmol/L)*	3.322 (1.359, 7.639)	0.008
*C-Reactive Protein (mg/L)*	1.012 (0.988, 1.036)	0.338
*D-Dimer (μg/L)*	1.052 (0.914, 1.212)	0.480

**Table 5:** Predictive assessment of combined serum MDA, infarct volume, and LDL levels for poor prognosis in patients with ACI

	AUC	*P* value	95 % Confidence Interval (*CI)*	Cut-off Value (ng/ml)	Sensitivity	Specificity
*MDA*	0.768	<0.05	(0.682, 0.855)	22.25	52.4 %	87.3 %
*Infarct Volume*	0.759	<0.05	(0.620, 0.809)	6.82	57.1 %	78.9 %
*Low-Density Lipoprotein*	0.614	<0.05	(0.506, 0.721)	2.87	52.4 %	66.2 %
*Combined Indicators*	0.784	<0.05	(0.692, 0.876)	0.36	69.0 %	81.7 %

## Abbreviations

GSH: glutathione
MDA: malondialdehyde
ACI: Acute cerebral infarct
ROS: reactive oxygen species
LOOH: lipid hydroperoxides
GSH: Glutathione
GPX4: Glutathione peroxidase 4
HIS: Hospital Information System
LIS: Laboratory Information Management System
PACS: Picture archiving and communication systems
NIHSS: National Institute of Health Stroke Scale
mRS: Modefied Rankin Scale
AUC: Area under curve
